# Threshold effects of body mass index on cognitive function and heterogeneity by sex and cardiovascular risk factors

**DOI:** 10.3389/fpubh.2022.897691

**Published:** 2022-07-19

**Authors:** Young-Joo Kim, Hyun-E Yeom

**Affiliations:** ^1^Department of Economics, Hongik University, Seoul, South Korea; ^2^Department of Nursing, Chungnam National University, Daejeon, South Korea

**Keywords:** aging, body mass index, cognitive impairment, cardiovascular disease, threshold

## Abstract

**Background:**

Disclosing the underlying relationship between body mass index (BMI) and cognitive decline is imperative for cognitive impairment prevention and early detection. Empirical studies have indicated the risk of abnormal BMI leading to cognitive impairment. However, the relative risk of underweight or overweight on cognitive function is obscure. This study investigated the asymmetric causal effect of BMI on cognitive decline below and above an unknown threshold and the heterogeneity in the threshold level and the magnitude of the threshold effect due to sex and cardiovascular risk factors.

**Methods:**

This study used 2010–2018 panel data from the Korean Longitudinal Study of Aging that assessed sociodemographic and health-related trends in Korean middle-aged to older population. A generalized method of moments estimator of the panel threshold model was applied to estimate the potential nonlinear pattern between BMI and cognitive function.

**Results:**

There was a threshold effect in the relationship between BMI and cognitive function. An increase in BMI below the threshold was associated with higher cognitive function, whereas a further increase in BMI above the threshold led to cognitive decline. The nonlinear pattern between BMI and cognitive function differed by sex and cardiovascular risk appearing more distinctively within men or the cardiovascular risk group.

**Conclusions:**

The detrimental impact of being underweight or overweight on cognitive function is heterogeneous by sex or cardiovascular risk. For obese men or individuals with cardiovascular risk factors, maintaining adequate BMI should be highlighted to help prevent cognitive decline.

## Introduction

Owing to the growing population of older adults worldwide, maintaining mental health in the aging population has become a priority. Cognitive impairment, including dementia, is a critical condition that threatens the quality of later life and increases the societal burden on long-term caregiving. According to a recent report by the World Health Organization (1), approximately 5% of the older adult population worldwide in 2015 was affected by dementia. Moreover, East Asian countries are rapidly transitioning to an aging society. As a result, they have been recognized as facing increasing trends in the prevalence of people living with cognitive impairments (2). For example, in Korea, the prevalence of dementia among adults aged 65 and older was 4.8% in 2010 and 8.1% in 2015, which increased to 11.2% in 2019 (3).

Numerous studies provide evidence on the link between body mass index (BMI) and cognitive function, mainly focusing on the elevated risk of cognitive decline from obesity. A longitudinal study by Gunstad et al. (4) found that individuals with higher BMI presented more deficient cognitive capabilities in memory, language, and global cognitive function. In addition, recent longitudinal studies (5, 6) revealed the long-term deleterious impact of obesity on cognitive function with evidence of a greater risk for dementia in later life for people who were obese in midlife. However, many other studies report the reverse relationship between BMI and cognitive function, indicating the uncertain and ambiguous relationship for their link. For instance, some studies (7–9) found that higher BMI, even above the obesity cutoff, was associated with a lower risk of cognitive decline, particularly in late-life, not midlife people. Other studies (9–11) found that long-term measures of BMI outside the normal range indicated by being either obese or underweight were associated with lower cognitive function in later life.

Furthermore, previous research notes that the influence of BMI on cognitive function varies according to underlying health conditions and non-modifiable factors (e.g., age and sex). Specifically, the detrimental effect of obesity significantly emerges in the population with cardiovascular diseases (CVD), including type 2 diabetes, hypertension, heart disease, or cerebrovascular disease [e.g., (12–17)]. Additionally, a considerable number of studies found sex-related differences in the impacts of BMI changes across diverse age groups on the accelerated decline in cognitive function (18–21).

The seemingly contradicting findings across studies calls for a more sophisticated approach that considers not only observable and unobservable individual-level variations, such as comorbid health conditions and sex but also a potential nonlinear relationship between BMI and cognitive function. Thus, this study employed a new approach to obtain a complete picture of the underlying relationship between BMI and cognitive function. The new approach achieves the following. First, it controls for unobservable confounding factors by eliminating constant individual characteristics over time using longitudinal data from middle-aged to older adults. Second, it allows for a BMI threshold point, at which the effect of BMI on cognitive function changes.

In summary, we aimed to derive the causal effect of BMI on cognitive function, focusing on differences in how cognitive function evolves over BMI below and above an unknown BMI threshold point. In addition, we examined potential heterogeneity in the threshold effect by checking whether the relational patterns between BMI and cognitive function differed by sex and CVD risk factors and how the threshold point varied across groups.

## Methods

### Data source and sample

This study employed eight-year panel data derived from the Korean Longitudinal Study of Aging (KLoSA). The KLoSA participants include Korean adults aged 45 and over, randomly selected using a regionally stratified approach to ensure national representation. The KLoSA was constructed to explore socioeconomic, health-related trends, and other dimensions of a rapidly changing society with an aging population, mainly covering seven topics: demographics, family, health, employment, income, assets, subjective expectations, and quality of life. The KLoSA surveys have been conducted biennially through computer-assisted personal interviews, which allow tracing the characteristics of participants from baseline over time. The KLoSA data were de-identified and are publicly available through public repositories (https://survey.keis.or.kr/eng/klosa/klosa01.jsp). For the current study, we extracted eight-year panel data of the KLoSA 2010–2018 that include adults (*N* = 4,848; Men = 2,070; Women = 2,778) who had not been medically diagnosed with dementia as the target population.

### Measures

BMI was calculated based on weight (kg) and height (m) using the formula (kg/m^2^). Cognitive functioning was measured using the Korean version of the Mini-Mental State Examination (MMSE). The MMSE is a fundamental tool globally validated and used to screen for cognitive impairment, including dementia and mild cognitive impairment. The MMSE comprises 11 items addressing seven sections, including orientation of time, place, and person, memorization, attention and calculation, recall, language, and visual construction. A score was summed for each section, ranging from 0 to 30, with higher scores indicating better cognitive function. The MMSE score for normal cognitive function is 24 or above, and a score of 17 or less is considered a risk factor for dementia.

We classified the CVD risk factor group as individuals with hypertension, diabetes, heart disease, or cerebrovascular disease. Considering potential confounding factors affecting the relationship between BMI and cognitive function, we controlled for sociodemographic characteristics, including age, annual household income, and marital status (i.e., whether currently married and living with a spouse). We included an additional set of covariates reflecting health-related behavioral factors, which included the essential skills for managing fundamental physical needs in daily living, measured using the activities of daily living (ADL) checklist. Other covariates were general health status, social interactions focusing on the monthly frequency of regular meetings with friends, and physical activity regarding engagement in regular exercise (yes or no).

### Statistical model and estimation methods

We employed the first-differenced generalized method of moments (GMM) estimator of the panel threshold model developed by Seo and Shin (22) as an alternative approach to the conventional least squares estimator of the linear regression model. The panel threshold model allows for a change in the relationship between BMI and cognitive function at a certain threshold point rather than imposing a single relationship as in the linear model. Hence, the threshold analysis verifies any nonlinear relationship between BMI and cognitive function by detecting different effects of BMI above and below the BMI threshold. The first-differenced GMM estimator is a GMM analysis after the first-difference transformation in the panel framework. Thus, we can explicitly control for individual time-invariant characteristics associated with BMI and cognitive function in estimating the relationship between BMI and cognitive function. Additionally, this approach is useful for evaluating an underlying threshold point wherein the relationship between BMI and cognitive function may change, as it also estimates the unknown threshold point.

The panel threshold model of this study is as follows:


(1) $$\begin{array}{l} y_{it}=\alpha_{0}+x_{1it}\alpha_{1}+\cdots +x_{kit}\alpha_{k}+BMI_{it}\vartheta +\delta \left(BMI_{it}-\gamma \right)\;\\ \;1\left\{BMI_{it}\geq \gamma \right\}+\omega_{i}+\varepsilon_{it}. \end{array}$$

We picked the 3rd, 5th, and 7th surveys from an eight-year period, 2010–2018, of the KLoSA. Therefore, we refer to these survey points as *t* = 3, 5, and 7. For an individual *i* at each period *t* (*t* = 3, 5, 7), *y*_*it*_ is MMSE score, and *x*_1*it*_ through *x*_*kit*_ are time-varying individual characteristics associated with cognitive function. ω_*i*_ is an individual fixed effect considered constant over time, and ε_*it*_ is an error term. γ is an unknown BMI threshold. Hence, for BMI below the threshold, ϑ is the BMI effect on cognitive function, and for BMI equal to and above the threshold, ϑ+δ is the total effect with the additional BMI effect δ. We impose the kink specification of (*BMI*_*it*_ − γ)1{*BMI*_*it*_ ≥ γ} to ensure that the change in BMI effect occurs continuously across the BMI threshold.

The estimation procedure is as follows. First, we format the dataset into a panel framework, stacking individual characteristics by time and removing all individuals with missing observations to derive balanced panel data. Second, we use the STATA command “*xthenreg*” developed by Seo et al. (23), which implements the first-differenced GMM estimator originally proposed and developed by Seo and Shin (22). Using this command, we specify BMI as a threshold variable and determine the covariates. Third, between continuous and discontinuous changes in the BMI impact at an unknown threshold, we choose a kink option for a continuous change in the BMI impact at the threshold. Fourth, we use a static model rather than the default dynamic model, as we do not consider lagged cognitive function as an explanatory variable. Finally, the GMM estimation with the first-difference transformation is implemented through the “*xthenreg*” command to estimate the unknown threshold (γ) and the BMI effects below the threshold (ϑ) and above the threshold (ϑ + δ).

For comparison, we also present the estimated BMI effects from two linear models: pooled OLS and fixed effects estimators. The pooled OLS is a conventional least squares method applied to panel data ignoring the panel setup. The fixed effects estimator is a linear regression approach accounting for individual time-invariant characteristics (i.e., fixed effects) with individual-specific time-demeaning. We are able to control for omitted variables that vary across individuals but do not change over time with fixed effects estimator. For example, gender and genetic factors are likely associated with both BMI and cognitive function but are constant over time. These individual's constant characteristics will be controlled for by individual-specific demeaning from the estimation model (1). The panel threshold estimation method is a more general model that takes account of individual's constant characteristics and incorporates nonlinearity in the BMI impact.

### Ethics approval and consent to participate

The surveys of the KLoSA were carried out after acquiring written informed consent from participants, and all procedures were conducted under relevant guidelines and regulations to protect the participants' anonymity. The current study received approval from the Institutional Review Board of Chungnam National University (No. 202111-SB-246-01).

## Results

### Descriptive characteristics of the sample

Table 1 presents the descriptive characteristics of the variables for each survey year used in this study. About 42.7% of the sample is men and 57.3% is women. The average MMSE score was 26.3 in 2010 and gradually decreased to 25.1 in 2018. However, the average BMI was stable at 23.4 over the 8-year survey period. Social interaction and physical activities were reduced over time as the monthly frequency of meeting friends and the proportion of individuals doing regular exercise decreased by 15 and 8%, respectively. In addition, the proportion of individuals who perceived good health changed from 37.6 to 26.05% between 2010 and 2018, respectively. For the same periods, the proportion of individuals who lived with a married spouse changed from 83 to 76%.

**Table 1:** Descriptive characteristics of the KLoSA participants.

**Variables**	**Year 2010**	**Year 2014**	**Year 2018**
	**M** ±**SD or**	**M** ±**SD or**	**M** ±**SD or**
	***n*** **(%)**	***n*** **(%)**	***n*** **(%)**
Age	62.623 (8.948)	66.623 (8.948)	70.623 (8.948)
ADL	0.032 (0.401)	0.049 (0.496)	0.119 (0.787)
BMI	23.410 (2.636)	23.405 (2.665)	23.407 (2.694)
MMSE Score	26.368 (4.117)	25.914 (4.602)	25.127 (5.446)
Household income^†^	7.492 (1.031)	7.499 (1.014)	7.582 (0.889)
Monthly frequency of meeting friends	7.200 (6.292)	6.352 (5.936)	6.126 (6.036)
General health condition ^§^^a^	1,824 (37.62)	1,424 (29.37)	1,263 (26.05)
Married^§^^b^	4,020 (82.92)	3,868 (79.79)	3,667 (75.64)
Regular exercise^§^^c^	1,765 (36.41)	1,649 (34.01)	1,624 (33.50)
Years of education	9.342 (3.349)	9.342 (3.352)	9.352 (3.354)

*Sample size is 4,848 individuals in each survey year*.

M±SD: Mean ± standard deviations, n (%): Number of observations (percentage).

† *Household income is logarithm of household income in 10,000*.

§ *Dummy variable reference groups*:

a *=have a good health status*;

b *= currently married and live with spouse*;

c *=exercise regularly*.

### Comparison of BMI effects on cognitive function by cross-sectional, longitudinal, and threshold estimation approaches

**Figure 1:**
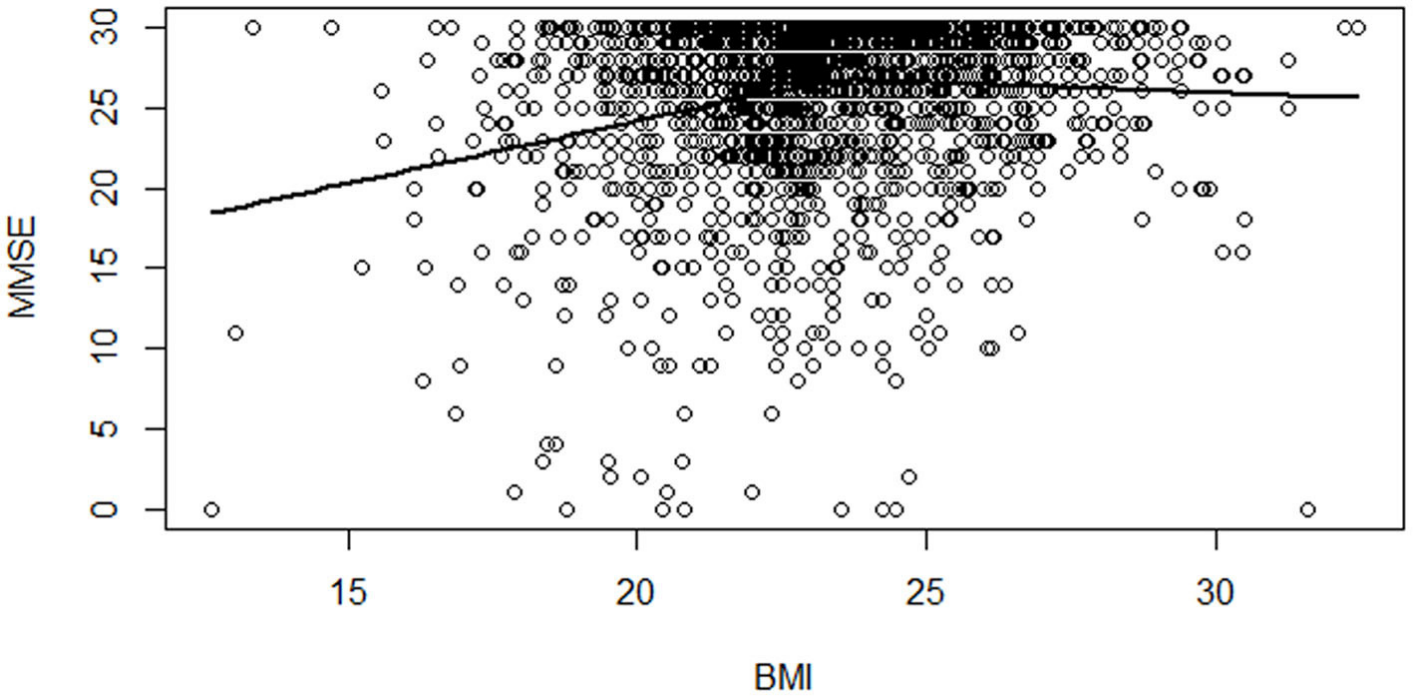
Scatterplot of MMSE scores vs. BMI among men with a fitted regression line. Notes: Each dot shows an individual's MMSE score residual and BMI for men in 2018. The solid line is the kink regression line fitted to capture the nonlinear relationship between MMSE and BMI.

**Figure 2:**
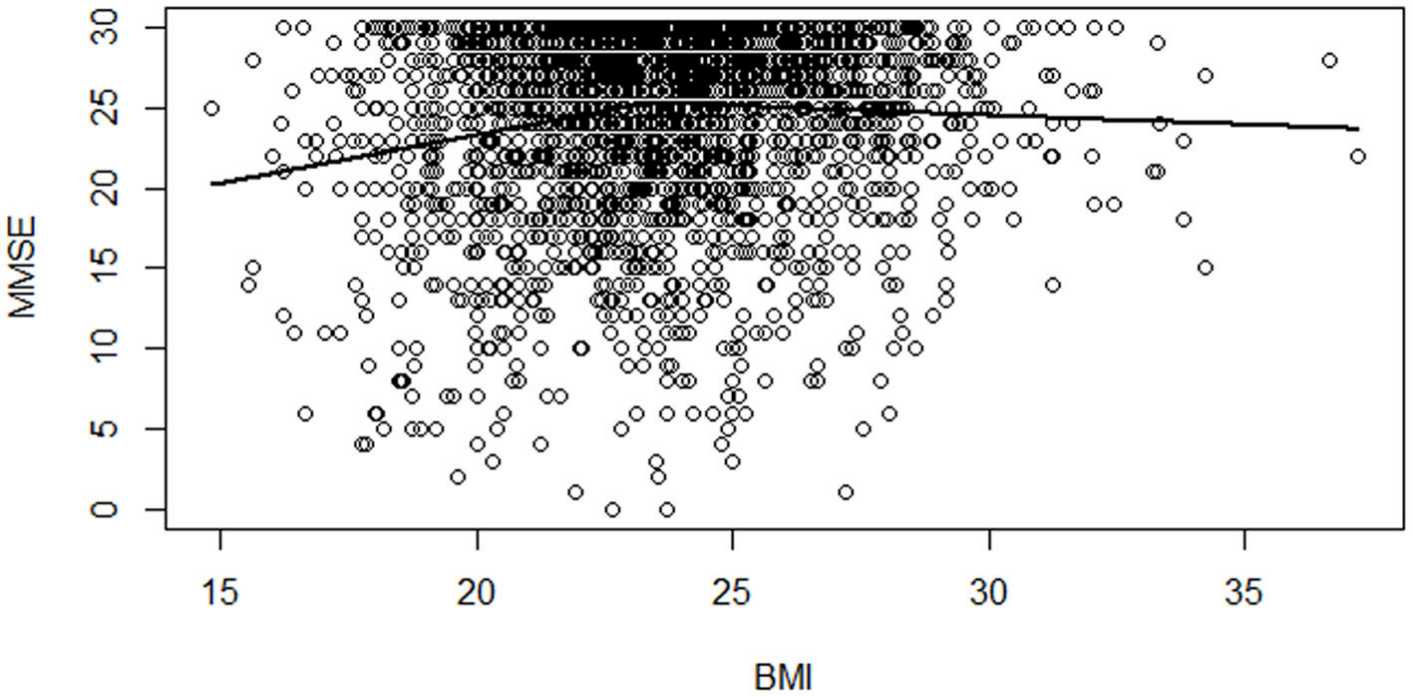
Scatterplot of MMSE scores vs. BMI among women with a fitted regression line. Notes: Each dot shows an individual's MMSE score residual and BMI for women in 2018. The solid line is the kink regression line fitted to capture the nonlinear relationship between MMSE and BMI.

As a preliminary examination, we illustrate the relationship between cognitive function and BMI with measures from 2018. We first regress MMSE scores with covariates listed in Table 1 and plot the residuals of MMSE against BMI for men and women, respectively in Figures 1, 2. In addition to the scatterplot of MMSE and BMI, we present fitted lines from the kink regression of MMSE on BMI. We can see that BMI is positively associated with MMSE for BMI up to the threshold, and then the relationship turns negative for BMI greater than the threshold. Other than the fitted lines, there is also heterogeneity in the variation of MMSE scores according to BMI. For BMI greater than a certain threshold, variation of MMSE scores becomes smaller.

Using the full sample, we employ the panel threshold method to formally account for unobserved individual's time-invariant characteristics and other confounding factors that are associated with cognitive function and BMI. We also adopt the pooled OLS and the linear panel fixed effects estimator for comparison.

Table 2 provides the estimated BMI effects on cognitive function according to the three approaches. First, the estimated impact of BMI on cognitive function from pooled OLS regression was statistically significant, demonstrating that a one-unit increase in BMI was associated with an increase in cognitive function score by 0.061. For pooled OLS, we also controlled for gender and years of education in addition to other time-varying regressors as presented in the last two rows. Second, the regression model based on the fixed effects method revealed a statistically significant impact of BMI on cognitive function. The estimated coefficient indicates that a one-unit increase in BMI is associated with an increase in cognitive function by 0.077.

**Table 2:** BMI effect on cognitive function in linear and non-linear models.

	**BMI effect on cognitive function**		
	**Pooled OLS**	**Fixed effects**	**Panel threshold**
	**ϑ (*SE*)**	**ϑ (*SE*)**	**γ, ϑorδ (*SE*)**
BMI threshold (γ)	N/A	N/A	23.234^***^(0.719)
Additional BMI effect over threshold (δ)	N/A	N/A	−8.000^***^ (2.527)
BMI effect (ϑ)	0.064^***^ (0.012)	0.077^**^ (0.031)	4.415^***^ (1.613)
Age	−0.148^***^ (0.004)	−0.129^***^ (0.008)	−0.105^***^(0.014)
ADL	−1.853^***^ (0.057)	−1.387^***^ (0.061)	−1.154^***^ (0.150)
Friends	0.017^***^ (0.005)	0.010 (0.006)	0.001 (0.011)
Good health	0.600^***^ (0.077)	0.419^***^ (0.081)	0.476^***^ (0.126)
Household income	0.239^***^ (0.039)	−0.044 (0.057)	0.006 (0.095)
Married	0.815^***^ (0.089)	0.238 (0.191)	−0.005 (0.374)
Regular exercise	0.991^***^ (0.070)	0.388^***^ (0.079)	0.252^**^ (0.127)
Years of education	0.189^***^ (0.012)		
Male	0.738^***^ (0.072)		
*R^2^*	0.324		

*Sample size is 14,544 from 4,848 individuals. Standard errors are in parentheses. Dependent variable is MMSE score. The pooled OLS regression model also includes indicators of gender and education level*.

*** *p < 0.01*,

** *p < 0.05*.

Lastly, the results of the panel threshold method present a different picture from those of the two linear model estimators. The estimates in column 3 show a nonlinear relationship between BMI and cognitive function with an estimated BMI threshold of 23.2. BMI is positively associated with cognitive function, such that a one-unit increase in BMI is associated with a 4.4 higher score of cognitive function. However, a further increase in BMI above the estimated threshold adds an impact of −8.0 to the initial impact of 4.4, resulting in an overall negative BMI impact on cognitive function. Notably, the BMI threshold of 23.2, as well as the BMI impacts below and above the threshold are significantly different from zero, supporting the notion of nonlinearity between BMI and cognitive function.

### Heterogeneous impacts of BMI on cognitive function by sex and CVD risk factors

Next, we divided the sample by sex and the presence of CVD risk factors to explore the relationship between BMI and cognitive function in a more homogenous group. Table 3 displays the effects of BMI on cognitive function according to sex and CVD risk factors. The upper panel shows the estimation results based on sex. The first row shows significant BMI threshold points for men and women, respectively. However, the BMI effects listed in the second and third rows present different patterns of BMI impact by sex.

**Table 3:** Threshold effect of BMI on cognitive function by groups.

	**BMI effect on cognitive**	
	**function by sex**	
	**Men**	**Women**
	**(*****n*** = **2,070)**	**(*****n*** = **2,778)**
	γ, ϑ* or δ* (*SE*)	γ, ϑ* or δ* (*SE*)
BMI threshold (γ)	23.681^***^(0.557)	22.937^***^(2.296)
BMI effect (ϑ)	3.186^***^ (0.977)	1.163 (1.633)
Additional BMI effect over threshold (δ)	−6.772^***^ (1.927)	−1.851 (2.150)
	**BMI effect on cognitive**	
	**function by CVD risk group**	
	**CVD Risk**	**No CVD Risk**
	**(*****n*** = **2,774)**	**(*****n*** = **2,074)**
	γ, ϑ* or δ* (*SE*)	γ, ϑ* or δ* (*SE*)
BMI threshold (γ)	23.657^***^(1.164)	20.889^***^(0.925)
BMI effect (ϑ)	4.260^***^ (1.347)	2.939 (2.083)
Additional BMI effect over threshold (δ)	−7.879^**^ (3.222)	−3.688 (2.381)

*Standard errors are in parentheses. Dependent variable is MMSE score. All models include controls of age, ADL, regular exercise, meeting with friends, household income, and marital status*.

*CVD Risk group: individuals with at least one incidence of hypertension, diabetes, heart disease, and cerebrovascular disease in the 3rd, 5th, or 7th survey*.

*** *p < 0.01*,

** *p < 0.05*.

The nonlinear relationship between BMI and cognitive function with a change in the BMI impact across the threshold point was clearly observed among men. A one-unit increase in BMI was positively associated with cognitive function (ϑ = 3.186,^***^p < 0.01) up to the BMI threshold (γ = 23.68,^***^p < 0.01). A further increase in BMI above this threshold was negatively associated with cognitive function (δ = -6.772, *p* < 0.01). For women, the threshold point was statistically significant (γ = 22.937,^***^p < 0.01), but both the BMI effects above and below the threshold were negligible.

When the sample was divided by CVD risk factors in the lower panel of Table 3, a statistically significant nonlinear impact of BMI emerged among those with CVD risk factors. The estimates in the next two rows show that the nonlinear BMI effect is evident only in the CVD risk group, although BMI threshold points were estimated from each subsample of the CVD risk group (γ = 23.657,^***^p < 0.01) and no-CVD risk group (γ = 20.889,^***^p < 0.01). For the CVD risk group, a one-unit increase in BMI up to the threshold (γ = 23.657) is positively associated with cognitive function (ϑ = 4.260,^***^p < 0.01). A further increase in BMI above the threshold has an additional BMI impact (δ = −7.879,^***^p < 0.01) on cognitive function, resulting in an overall negative BMI impact on cognitive function. In contrast, such a significant BMI impact, either linear or nonlinear, was not observed in the no-CVD risk group.

### The joint impact of sex and CVD risk factors in the relationship between BMI and cognitive function

The empirical findings of the relationship between BMI and cognitive function considering the combined effects of sex and CVD risk factors are displayed in Table 4. Briefly, there were nonlinear relationships between BMI and cognitive function with a significant BMI threshold (γ) for men in general and specifically for CVD risk groups of men and women.

**Table 4:** Threshold effect of BMI on cognitive function by sex and CVD risk groups.

	**Men**		**Women**	
	**CVD risk**	**No CVD risk**	**CVD Risk**	**No CVD Risk**
	**(*****n*** = **1,159)**	**(*****n*** = **911)**	**(*****n*** = **1,615)**	**(*****n*** = **1,163)**
	γ, ϑ* or δ* (*SE*)	γ, ϑ* or δ* (*SE*)	γ, ϑ* or δ* (*SE*)	γ, ϑ* or δ* (*SE*)
BMI threshold (γ)	23.319^***^(0.822)	21.008^***^(1.093)	23.009^***^(0.910)	24.049^***^(2.221)
BMI effect (ϑ)	3.857^**^(1.568)	2.890^**^ (1.448)	3.595^*^(1.939)	−0.949 (0.736)
Additional BMI effect over threshold (δ)	−6.777^***^(2.286)	−3.560^**^ (1.533)	−5.540^**^ (2.728)	2.348 (1.429)

*Standard errors are in parentheses. Dependent variable is MMSE score. All models include controls of age, ADL, regular exercise, meeting with friends, household income, and marital status*.

*CVD Risk group: individuals with at least one incidence of hypertension, diabetes, heart disease, and cerebrovascular disease in the 3rd, 5th, or 7th survey*.

*** *p < 0.01*,

** *p < 0.05*,

* *p < 0.1*.

For men, the nonlinear relationship remains statistically significant regardless of the CVD risk factors. An increase in BMI was associated with higher cognitive function up to the threshold value (ϑ = 3.857, *p* < 0.05 for CVD risk group, ϑ = 2.890, *p* < 0.05 for non-CVD risk group). A further increase in BMI beyond the threshold was associated with decreased cognitive function (δ = −6.777 for CVD risk, δ = −3.560 for the no-CVD risk group).

For women, the nonlinear BMI effect on cognitive function was found only among those with CVD risk factors. An increase in BMI up to the threshold of 23 was associated with an increase in cognitive function (ϑ = 3.595, *p* < 0.1). However, a further increase in BMI entails a decrease in cognitive function (δ = −5.540, *p* < 0.01), resulting in an overall reduction in cognitive function. For women without CVD risk factors, the BMI effect was small and not statistically significant below and above the estimated threshold of 24.

When we focus on the CVD risk group, the BMI effect below the threshold is similar across men and women, but the size of the negative impact above the threshold is larger for men than women. In the group with no-CVD risk factors, significant BMI effects emerged only among men.

Another notable point from Table 4 is that in the CVD risk group, the BMI threshold was about 23 for both men and women; (γ = 23.319, *p* < 0.01) for men and (γ = 23.009, *p* < 0.01) for women. The BMI threshold is consistent between men and women once we focus on the group with CVD risk factors. In contrast, the BMI effect changes at a lower threshold for men without CVD risk factors (γ = 21.008, *p* < 0.01).

## Discussion

Identifying criterion factors linked to cognitive function is crucial for early detection and prevention of cognitive decline in an aging population. This study investigated the long-term relationship between BMI and cognitive function over 8 years, considering possible differences in the impact by sex and CVD risk factors. As a new approach, this study applied a panel threshold estimation method for detecting potential nonlinear and heterogeneous effects of BMI on cognitive function. Additionally, it estimated both the way how the impact of BMI on cognitive function changes across a certain BMI threshold and the unknown threshold.

This study makes a novel contribution to the literature by noting that the impact of BMI on cognitive function in middle-aged to older Koreans changes at a certain threshold point. That is, there is a nonlinear relationship between BMI and cognitive function. An increase in BMI was associated with higher cognitive function up to the estimated threshold. On the contrary, above the threshold, a further increase in BMI led to cognitive decline.

The significant effects of BMI observed in men or individuals with CVD risk factors on cognitive function align with previous studies reporting the relative risks on cognitive function among the elderly. For example, cognitive impairment has been documented to be more prevalent among individuals with various CVD risk factors, including type 2 diabetes (15), metabolic syndrome (13), or the accumulation of several risk factors (14, 17). Additionally, the impact of obesity on cognitive function was observed with a notably larger impact for men with hypertension (12).

After establishing that CVD risk factors are critical elements associated with cognitive decline, this study further extends the literature by demonstrating the nonlinear relationship between BMI and cognitive function among men and women with CVD risk factors. Furthermore, the sex-related difference in the risk of cognitive decline resulting from a change in BMI demonstrates that both men and women are likely to have cognitive decline due to an increase in BMI above the threshold, particularly in the presence of CVD risk factors.

This study also contributes to the literature by addressing the contradictory findings on the estimated link between BMI and cognitive function. Previous studies examining the aging population documented both BMI's protective and adverse impacts on cognitive function. Some studies found a protective effect of BMI increase on cognitive function (6, 24). In contrast, other studies found unfavorable, mixed, or no significant impact of BMI increase for different BMI ranges (25, 26). The findings of heterogeneous BMI effects varying by BMI value indicate the presence of asymmetric effects of BMI rather than a single impact in either the positive or negative form. Our findings support the vital role of BMI in cognitive function over the life course of older adults in the early stages of cognitive decline or preclinical dementia.

Finally, the estimated BMI threshold points, at which the adverse effects of BMI on cognitive function occurred, were around 23 for most groups. This threshold value corresponds to the cutoff point for evaluating obesity in the Korean population. As the findings were derived from longitudinal data of a nationally representative aging population, the discovery supports the obesity classification based on 23 by providing further evidence that BMI ≥ 23 is a valid gold standard for assessing obesity and classifying the most at-risk group of men and women vulnerable to cognitive decline and other related health problems in middle-aged to older Koreans.

This study has several implications. First, the significant impact of BMI on cognitive function derived from longitudinal data following individuals over 8 years showed that both protective (BMI ≤ threshold) and detrimental (BMI ≥ threshold) impacts of BMI can occur at all stages of mid to late-life depending on the BMI range. Second, this study identified those more vulnerable to cognitive decline from a change in BMI. Finally, our findings indicate that secure monitoring of BMI is more critical for men than women to avoid cognitive decline from an increase in BMI above the threshold.

In addition to the strengths discussed thus far, this study has limitations. First, we measured cognitive function with the MMSE. However, further analysis with specific domains of cognitive function, including memory and language skills, would be informative for drawing a detailed picture of the impact of BMI on cognitive decline. Second, despite the panel analysis, the study sample is from an 8-year observation among Koreans. For the generalizability of findings to global populations, further examination based on the threshold approach is needed using international samples. Third, this study considered a CVD risk factor as a health-related indicator that could affect the relationship between BMI and cognitive function. Although we adjusted for various confounding factors linked to comorbidity, further study with biomedical characteristics (e.g., blood pressure, glucose level) is suggested for early detection of individuals at higher risk of cognitive decline.

## Conclusion

Prevention and early detection of cognitive decline are critical concerns in the era of growing elderly population. This study identified threshold points of BMI where the impact of BMI on cognitive function changed and showed that the effects of BMI on cognitive function were different below and above the threshold, particularly for men or individuals with CVD risk factors. The findings from this study highlight useful assessment of BMI for groups of individuals who face higher risk of cognitive decline from a change in BMI above the threshold. Further investigation from worldwide global data is warranted to extend our findings across diverse ethnic and sociocultural groups.

## Data availability statement

Publicly available datasets were analyzed in this study. This data can be found here: https://survey.keis.or.kr/eng/klosa/klosa01.jsp.

## Ethics statement

The studies involving human participants were reviewed and approved by the Institutional Review Board of Chungnam National University (No. 202111-SB-246-01). The patients/participants provided their written informed consent to participate in this study.

## Author contributions

Y-JK conceptualized the research idea, analyzed data, interpreted the estimation results, and wrote the first version of the manuscript. H-EY conceived and designed the analysis, revised the manuscript, and supervised the project. Y-JK and H-EY contributed to writing the manuscript. Both authors read and approved the final manuscript.

## Funding

This work was supported by the Ministry of Education of the Republic of Korea and the National Research Foundation of Korea [NRF- 2020S1A5A2A03046422].

## Conflict of interest

The authors declare that the research was conducted in the absence of any commercial or financial relationships that could be construed as a potential conflict of interest.

## Publisher's note

All claims expressed in this article are solely those of the authors and do not necessarily represent those of their affiliated organizations, or those of the publisher, the editors and the reviewers. Any product that may be evaluated in this article, or claim that may be made by its manufacturer, is not guaranteed or endorsed by the publisher.
